# Supplementary material for: Conserved transcription factors promote cell fate stability and restrict reprogramming potential in differentiated cells
Source: Nat Commun. 2023 Mar 27;14:1709. doi: 10.1038/s41467-023-37256-8 (PMC10043290; doi:10.1038/s41467-023-37256-8)
Supplement: Supplementary file 1 — Supplementary Information [file 41467_2023_37256_MOESM1_ESM.pdf]

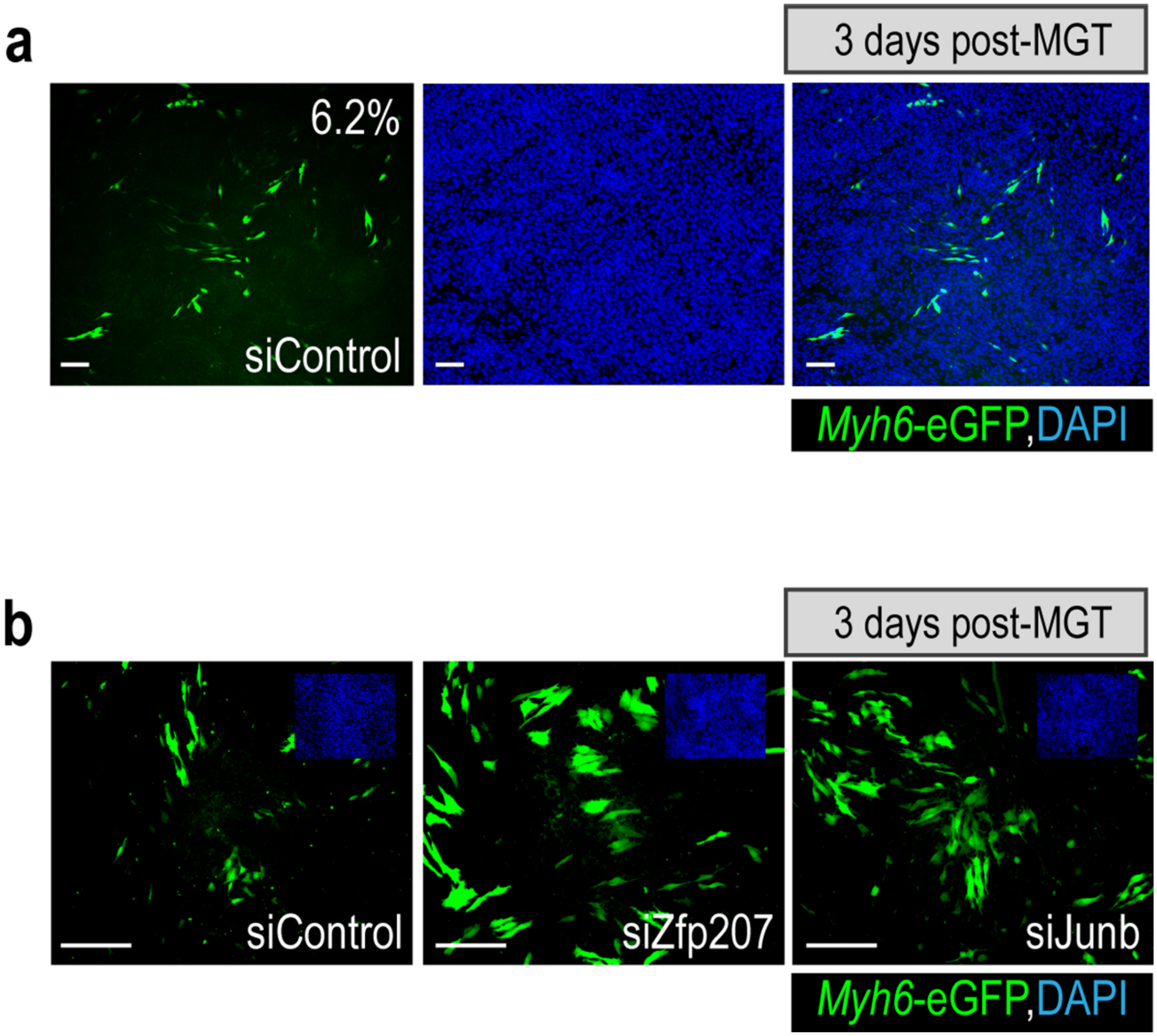

Supplemental Figure 1. (**a, b**) Representative images of siControl-transfected with average reprogramming efficiency quantification (6.2% of Myh6-eGFP+ cells) (**a**) and siControl-, siZfp207-, or siJunb-transfected (**b**) iMGT-MEFs 3 days after MGT overexpression. *Myh6-eGFP* is shown in green, and nuclei are stained with DAPI (blue). Scale bars: 50 µm. Groups were compared using two-tailed unpaired. N=4 per condition.

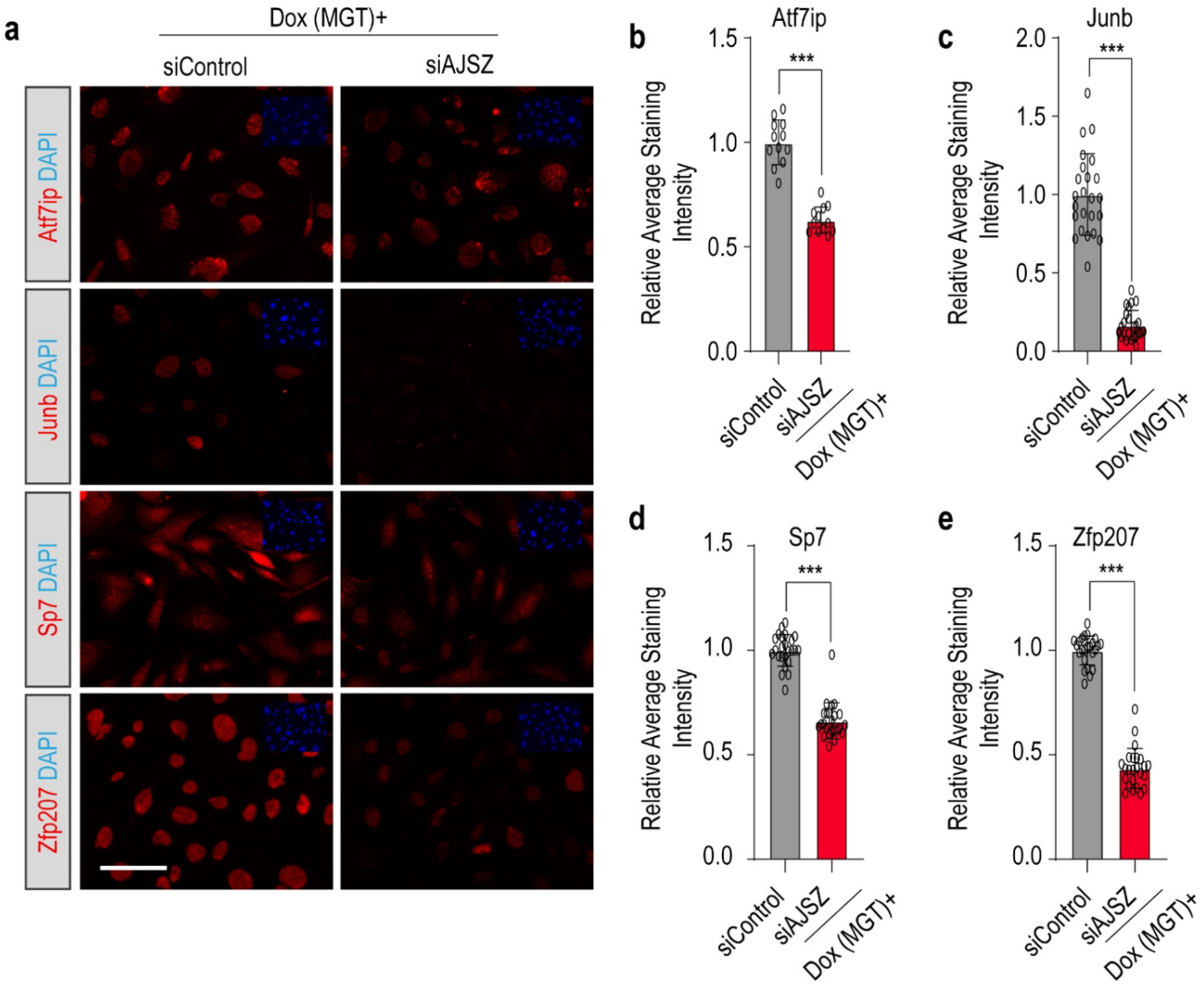

Supplemental Figure 2. (a) Representative immunofluorescence images of Atf7ip, Junb, Sp7 and Zfp207 protein levels (red) in response to siControl or siAJSZ, days 3 days post-transfection in iMGT-MEFs. DAPI is shown in blue. **(b-e)** Bar graph showing quantification of the average Atf7ip n=12 per condition (b), Junb n=24 per condition (c), Sp7 n=24 per condition (d) and Zfp207 n=24 per condition (e) staining intensity in response to siControl or siAJSZ transfection. ***P< 0.001. Scale bars: 50 µm. Data in figure are presented as mean values +/- standard deviation.

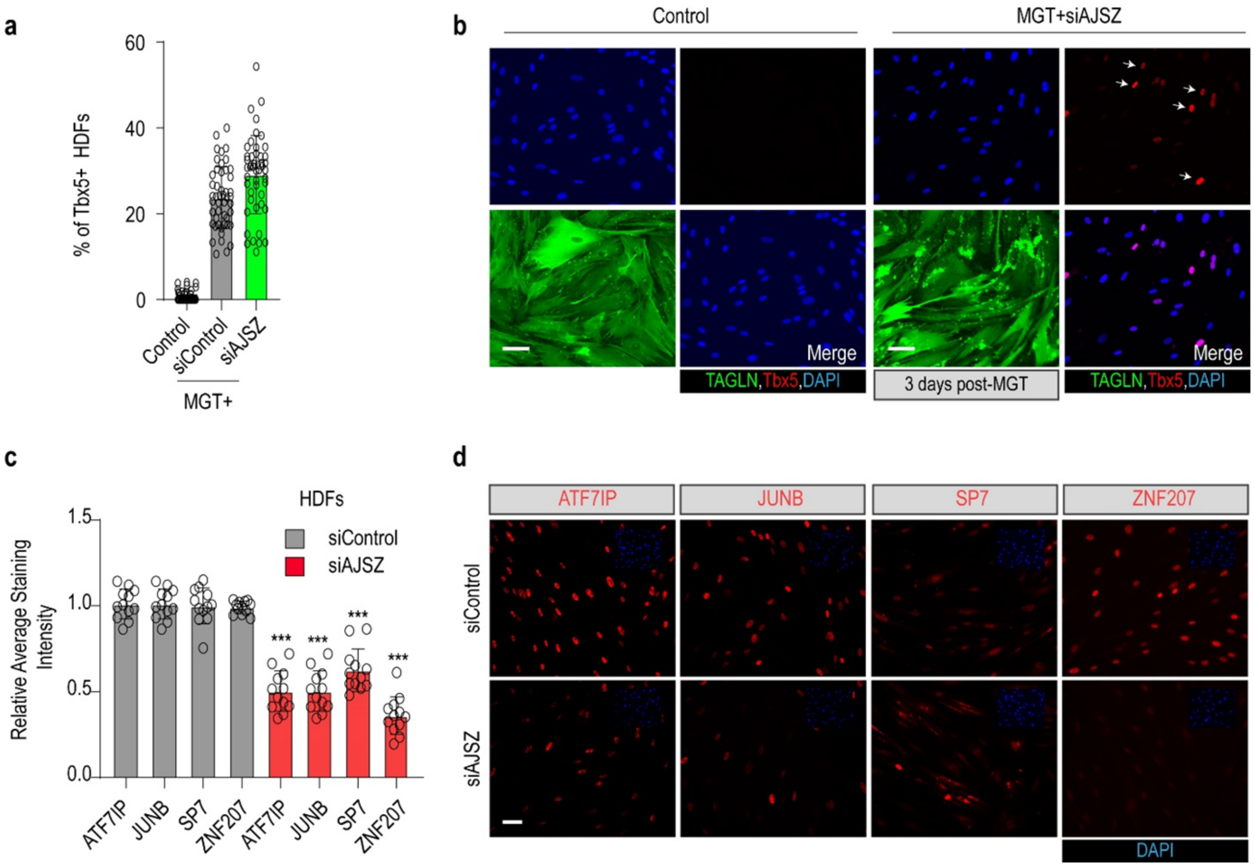

Supplemental Figure 3. (a) Quantification of the % of Tbx5 expressing HDFs, three days post retrovirus mediated MGT overexpression. N=48 per condition. **(b)** Representative immunofluorescence images of DAPI (nuclei), TAGLN (fibroblast marker, green) and TBX5 (Red) in control and MGT+siAJSZ conditions. **(c)** Bar graph showing quantification of the average ATF7IP, JUNB, SP7 and ZNF207 staining intensity in siControl vs siAJSZ conditions, three days post-transfection. N=12 per condition. ***P< 0.001. **(d)** Representative immunofluorescence images of ATF7IP, JUNB, SP7 and ZNF207 protein levels in siControl vs siAJSZ days 3 days post-transfection. DAPI is shown in blue. Scale bars: 50 µm. Data in figure are presented as mean values +/- standard deviation.

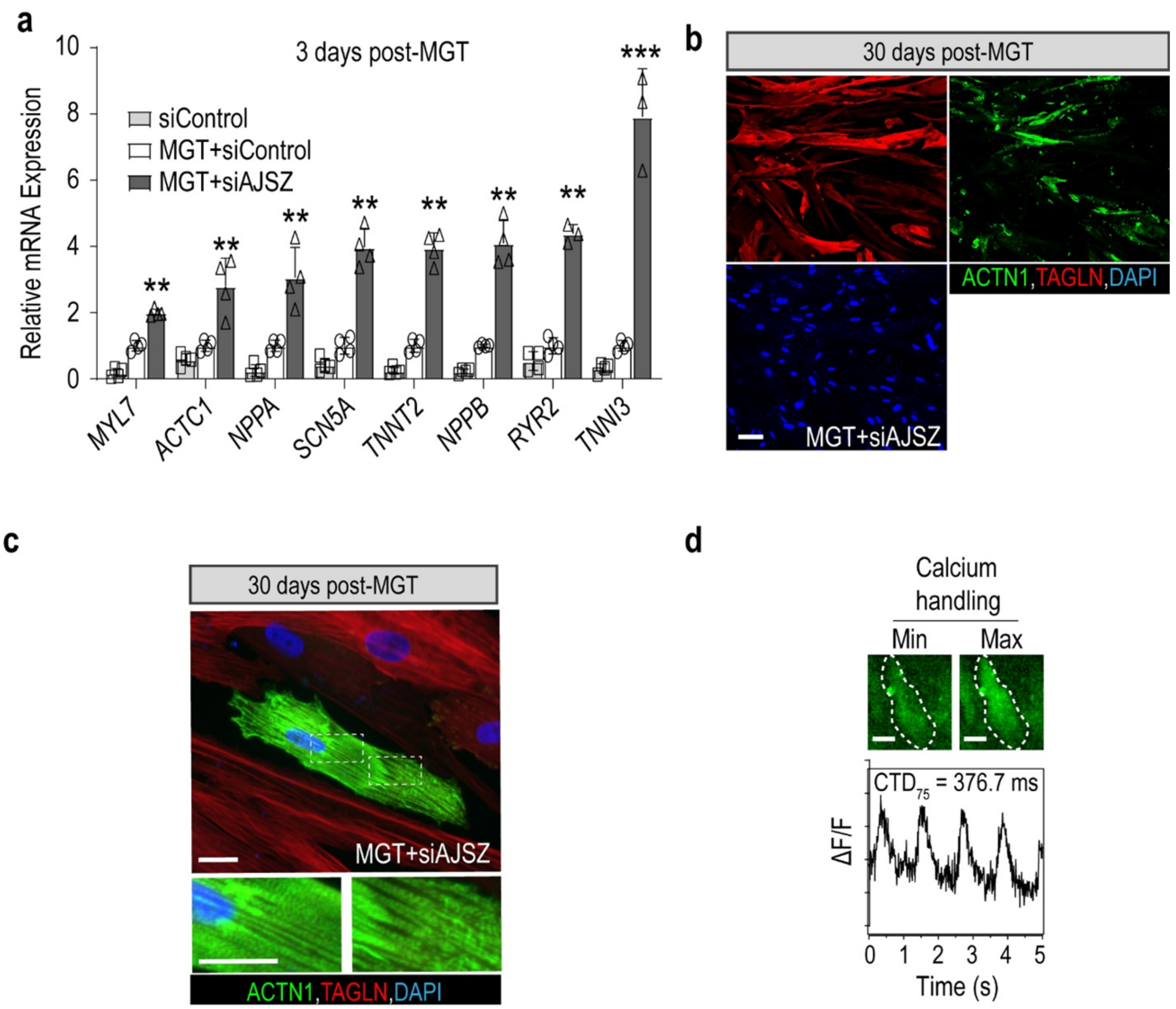

Supplemental Figure 4. (**a**) qRT-PCR analysis of AJSZ expression in siCTR- or siAJSZ-transfected HDFs 3 days after mMGT overexpression. Data was normalized to the MGT+siCTR cells. MYL7 n=6, ACTC1 n=10, NPPA n=11, SCN5A n=6, TNNT2 n=6, NPPB n=6, RYR2 n=2, TNNI3 n=2 per condition. Data are presented as mean values +/- standard deviation. (**b**) qRT-PCR analysis of the indicated cardiac gene expression in siCTR- or siAJSZ-transfected HDFs 3 days after mMGT overexpression. (**c**) Immunostaining of ACTN1 and TAGLN in siAJSZ-transfected HDFs analyzed 30 days after MGT overexpression. Lower panels show that some ACTN1$^+$ cells have lost TAGLN staining and show striations. (**d**) Fluorescence-based (Fluo-4) quantification of calcium handling in siAJSZ-transfected HDFs, 30 days after MGT overexpression. N=4 per

condition. Scale bars: 50 μm. Student's t-test. *p<0.05, **p<0.01, p***<0.001, ****p<0.0001. Groups were compared using two-tailed unpaired.

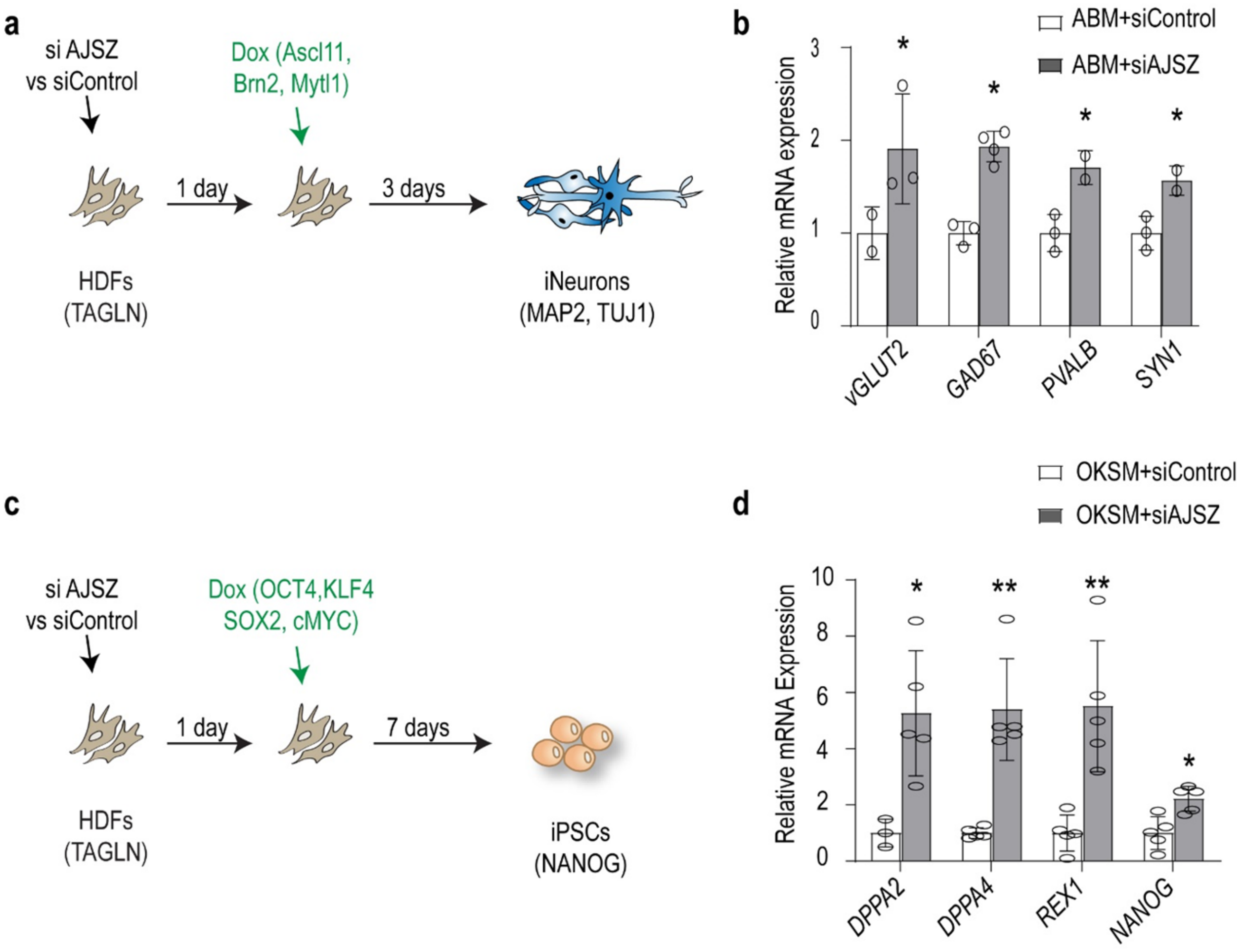

Supplemental Figure 5. (**a**) Schematic showing the experimental set-up for direct neuronal reprogramming of HDFs with ABM. (**b**) qRT-PCR analysis of neuronal markers in siRNA-transfected HDFs, 3 days after induction of neuronal reprogramming with ABM. vGLUT2 n=3, GAD67 n=4, PVALB n=3, SYN1 n=3 per condition. (**c**) Schematic showing the experimental set-up for reprogramming of HDFs into iPSCs with OKSM. (**d**) qRT-PCR analysis of pluripotent markers in siControl- and siAJSZ-transfected HDFs on day 7 after OKSM overexpression. DPPA2 n=3, DPPA4 n=5, REX1 n=5, NANOG n=5 per condition. Student's t-test. *$p<0.05$, **$p<0.01$, ***$p<0.001$, ****$p<0.0001$. Groups were compared using two-tailed unpaired. Scale bars: 50 µm. Data in figure are presented as mean values +/- standard deviation. (a) and (c) schematics are modified from Cunningham, T. J. et al. Id genes are essential for early heart formation. Genes & development, doi:10.1101/gad.300400.117 (2017). - CC-BY 4.0.

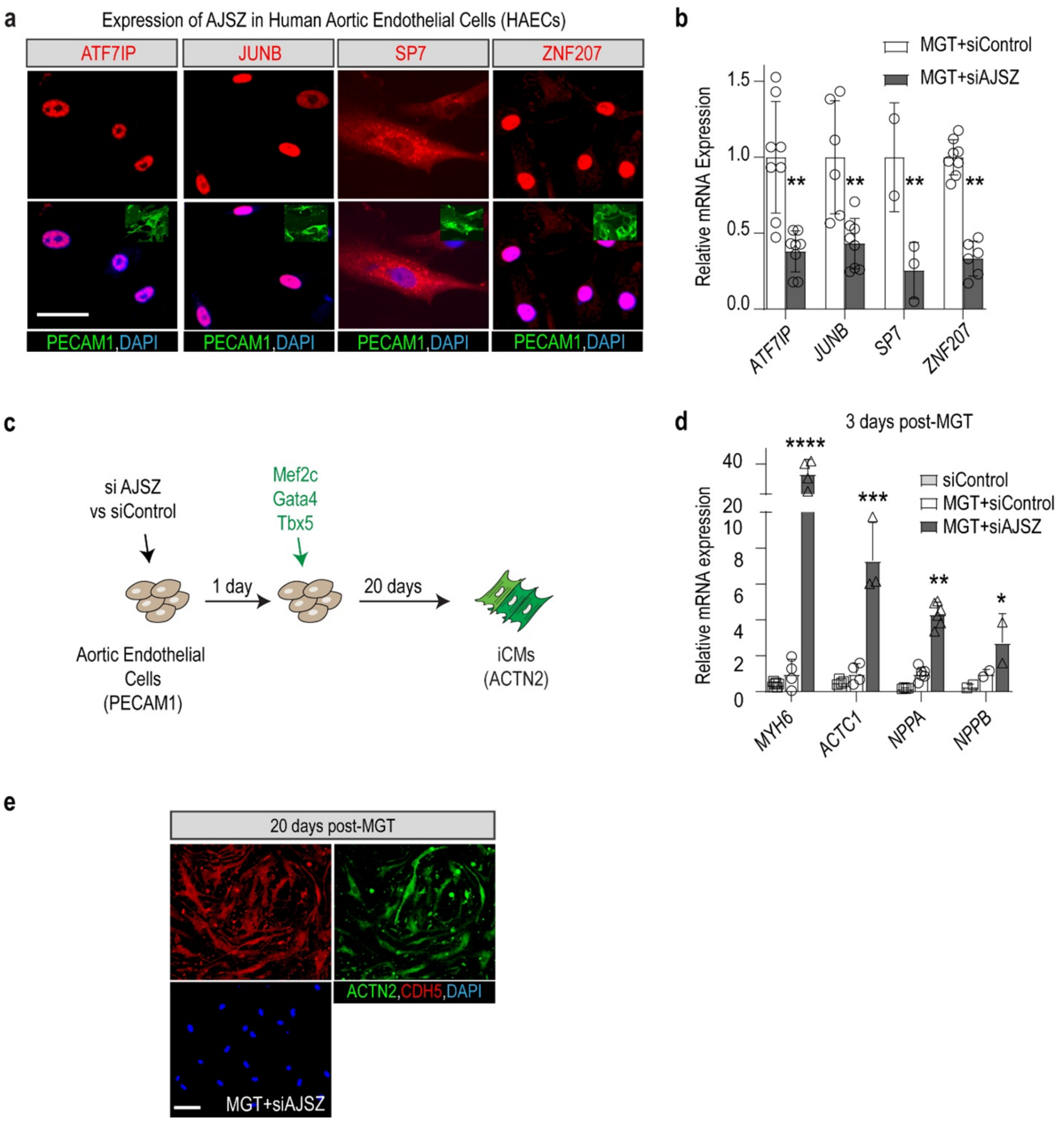

Supplemental Figure 6. (**a**) Immunostaining of ATF7IP, JUNB, SP7 and ZNF207 (red) and endothelial marker PECAM1 (green) in HAECs. Nuclei are stained with DAPI (blue, top left insets). Scale bars: 50 µm. (**b**) qRT-PCR analysis of AJSZ expression in siControl- and siAJSZ-transfected HAECs on day 2 after MGT overexpression. ATF7IP n=8, JUNB n=8, SP7 n=3, and ZNF207 n=8 per condition. (**c**) Schematic depicting direct cardiac reprogramming assay in HAECs. (**d**) qRT-PCR of cardiac genes in siControl- and siAJSZ-transfected HAECs on day 3

after MGT overexpression. MYH6 n=4, ACTC1 n=3, NPPA n=6, NPPB n=2 per condition. (**e**) Immunostaining of CDH5 (red =endothelial cell marker), ACTN2 (green =cardiac marker) and DAPI (blue) in HAECs 20 days post- MGT overexpression. Scale bars: 50 µm..Student's t-test. *p<0.05, **p<0.01, ***p<0.001, ****p<0.0001. Groups were compared using two-tailed unpaired. (c) schematic is modified from Cunningham, T. J. et al. Id genes are essential for early heart formation. Genes & development, doi:10.1101/gad.300400.117 (2017). - CC-BY 4.0.

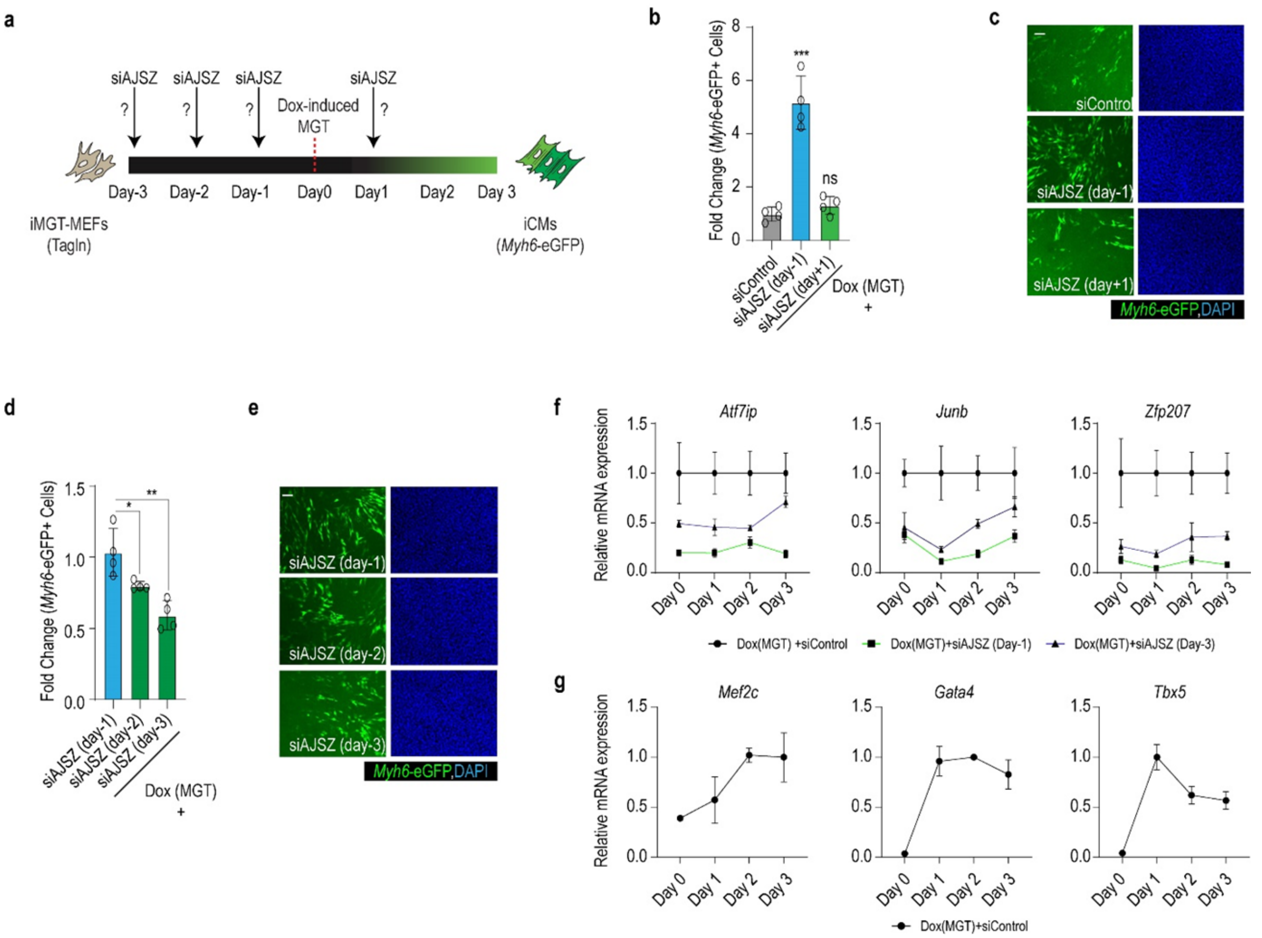

Supplemental Figure 7. (**a**) Experimental strategy to determine optimal AJSZ KD timing, to elicit maximal CR efficiency in iMGT-MEFs assay. (**b, c**) Quantification, n=4 per condition, (**b**) and representative images (**c**) of reprogramming efficiency in response to siAJSZ one day prior or one day after MGT overexpression. Data are presented as mean values +/- standard deviation. (**d, e**) Quantification, n=4 per condition, (**d**) and representative images (**e**) of reprogramming efficiency in response to siAJSZ 1, 2, or 3 days before induction of MGT. Data are presented as mean values +/- standard deviation. (**f**) Time course (day 0-3) of *Atf7ip*, *Junb* and *Zfp207* relative mRNA expression in Dox (MGT) +siControl, Dox (MGT)+siAJSZ (Day-1) and Dox (MGT)+siAJSZ (Day-3) conditions. N=3 per condition. Data are presented as mean values +/- SD. (**g**) Time course (day 0-3) of *Mef2c*, *Gata4* and *Tbx5* relative mRNA expression post-Dox treatment. N=3 per condition. Data are presented as mean values +/- standard deviation. two-way ANOVA, $*p<0.05$, $**p<0.01$, $***p<0.001$. Scale bars: 30 µm. (a) schematic is modified from Cunningham, T. J. et

al. Id genes are essential for early heart formation. Genes & development, doi:10.1101/gad.300400.117 (2017). - CC-BY 4.0.

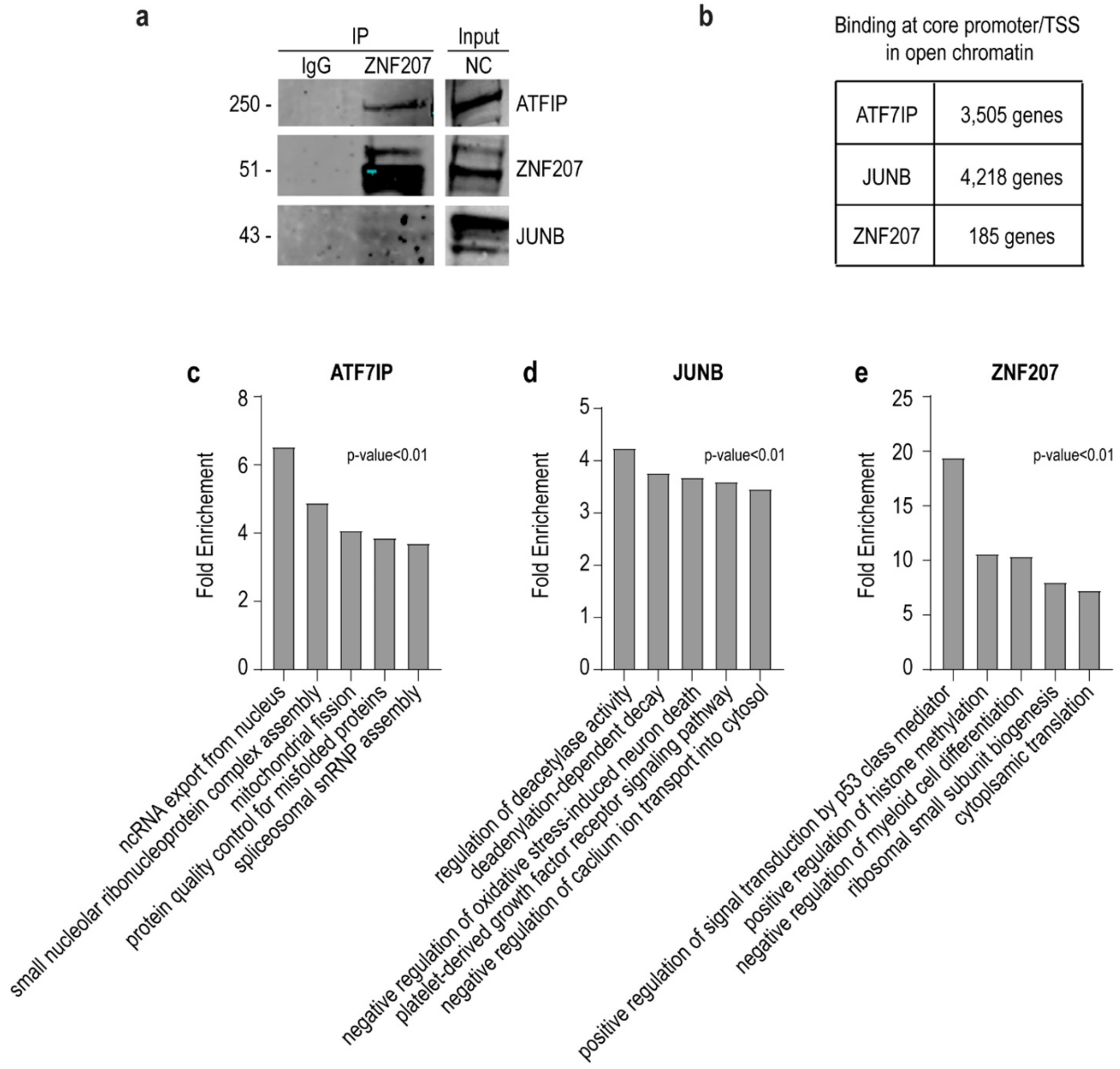

Supplemental Figure 8. (a) Western blot showing that ZNF207 co-immunoprecipitates ATF7IP and JUNB in HDFs. N=2 per condition. For full scan blots, see the Source Data file. (**b**) Table reporting the number of core promoters/TSS bound by ATF7IP, JUNB and ZNF207 in HDFs. (**c-e**) GO term analysis for genes bound by ATF7IP (c), JUNB (d), ZNF207 (e) at their core promoter regions.

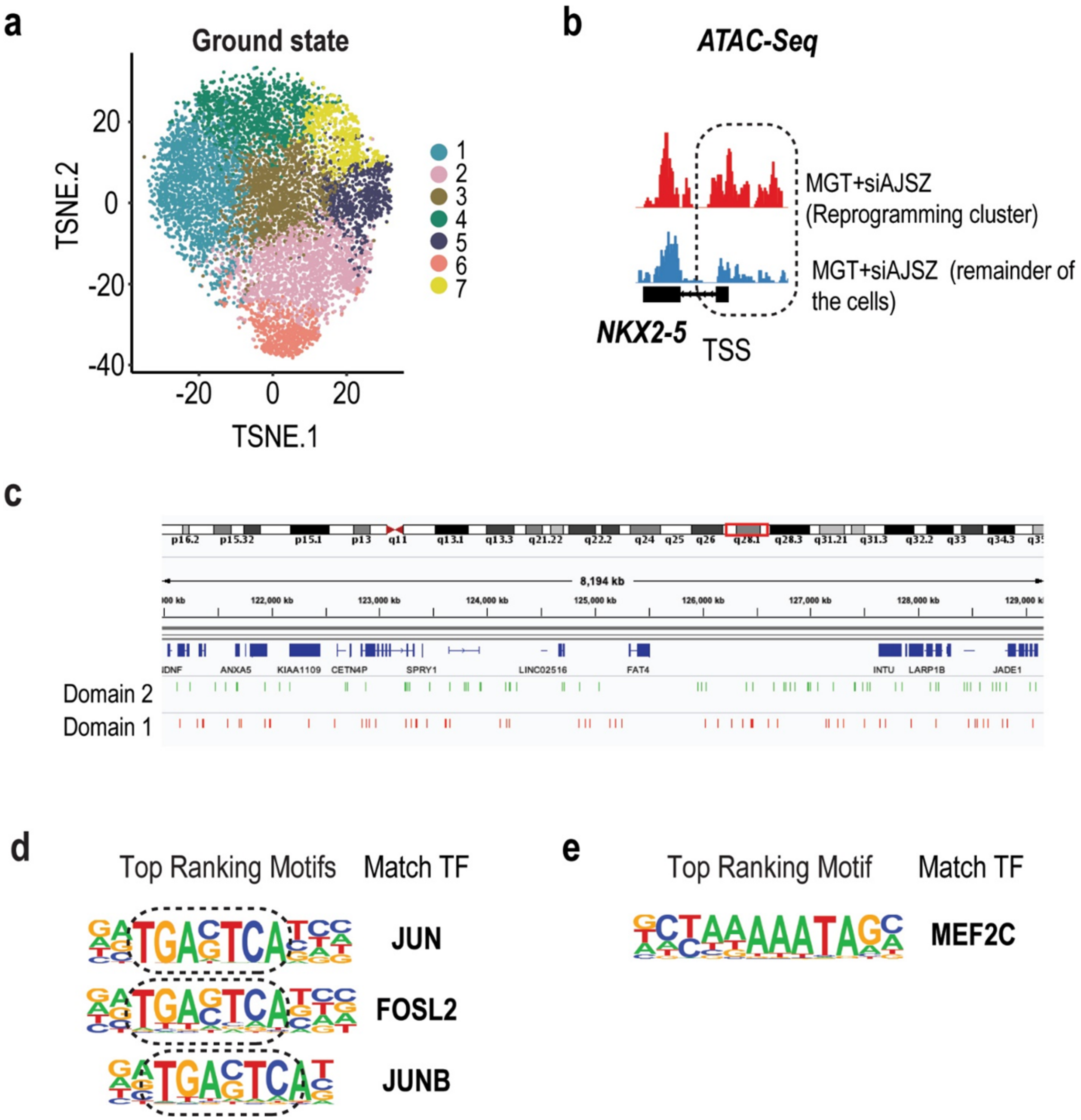

Supplemental Figure 9. (**a**) t-SNE visualization of cell clusters in HDFs at ground state using scATAC-seq. (**b**) ATAC-seq track for NKX2.5 at the TSS region in reprogramming cluster (cluster 2) as compared to the remainder of the cells. (**c**) Example of *domain 1* and *domain 2* distribution across ~8MB at region 4 q28.1. Domains 1 and 2 consist in evenly distributed short stretches of DA chromatin. (**d**) Top 3 motifs enriched in *domain 1* are AP-1 TF motifs. (**e**) Second most motif enriched in *domain 2* corresponds to reprogramming TF MEF2C putative DNA binding site.

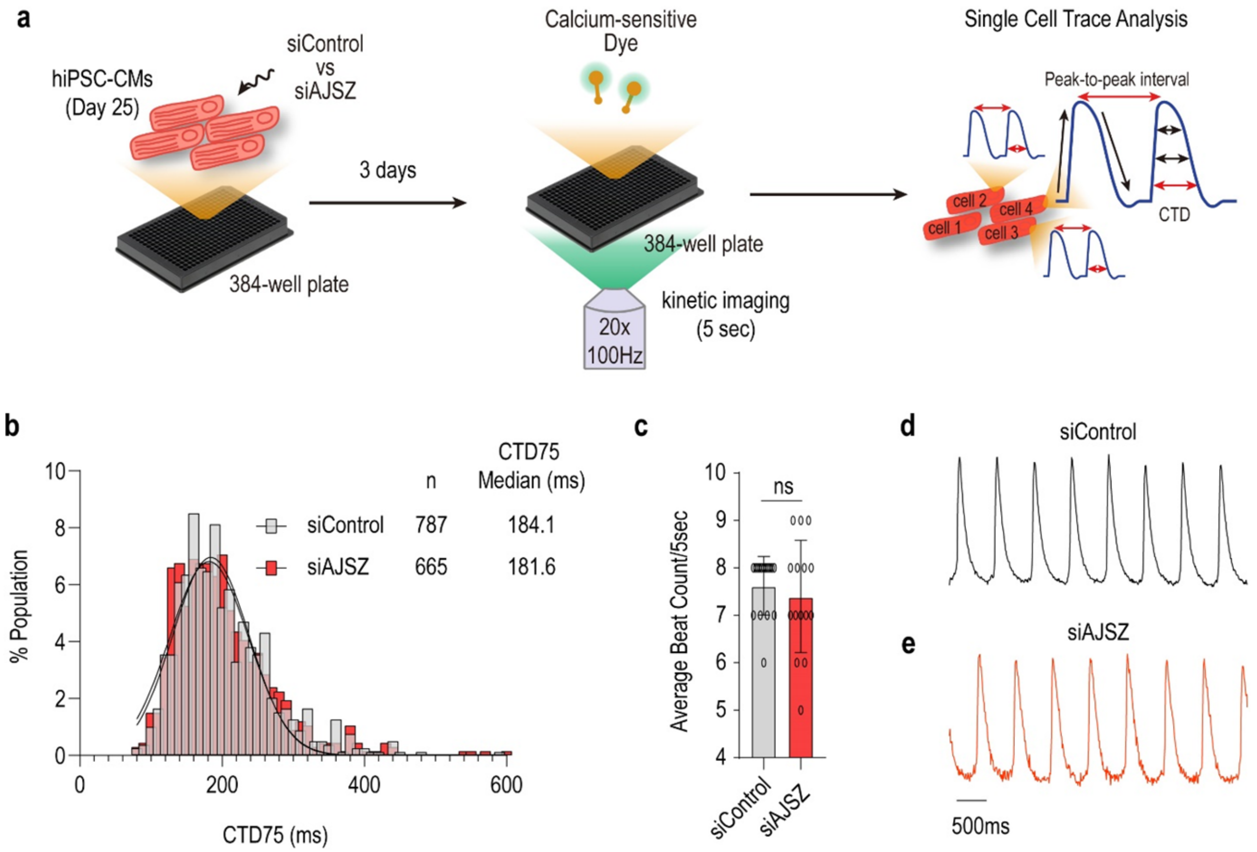

Supplemental Figure 10. (a) Schematic depicting experimental strategy to evaluate effect of siAJSZ on calcium handling in hiPSC-CMs. (**b**) Distribution of calcium transient duration values (CTD 75) from hiPSC-CMs, in siAJSZ and siControl conditions. **(c)** Bar graph showing the quantification of the average number of beats per 5 seconds in siControl vs siAJSZ condition. N=16 for siControl and N=15 for siAJSZ. Groups were compared using two-tailed unpaired. Data are presented as mean values +/- standard deviation. (**d,e**) Representative calcium transient peak trains for siControl (d) and siAJSZ (e). No effect of siAJSZ on calcium handling is observed as compared to siControl. (a) schematic is modified from Cunningham, T. J. et al. Id genes are essential for early heart formation. Genes & development, doi:10.1101/gad.300400.117 (2017). - CC-BY 4.0.

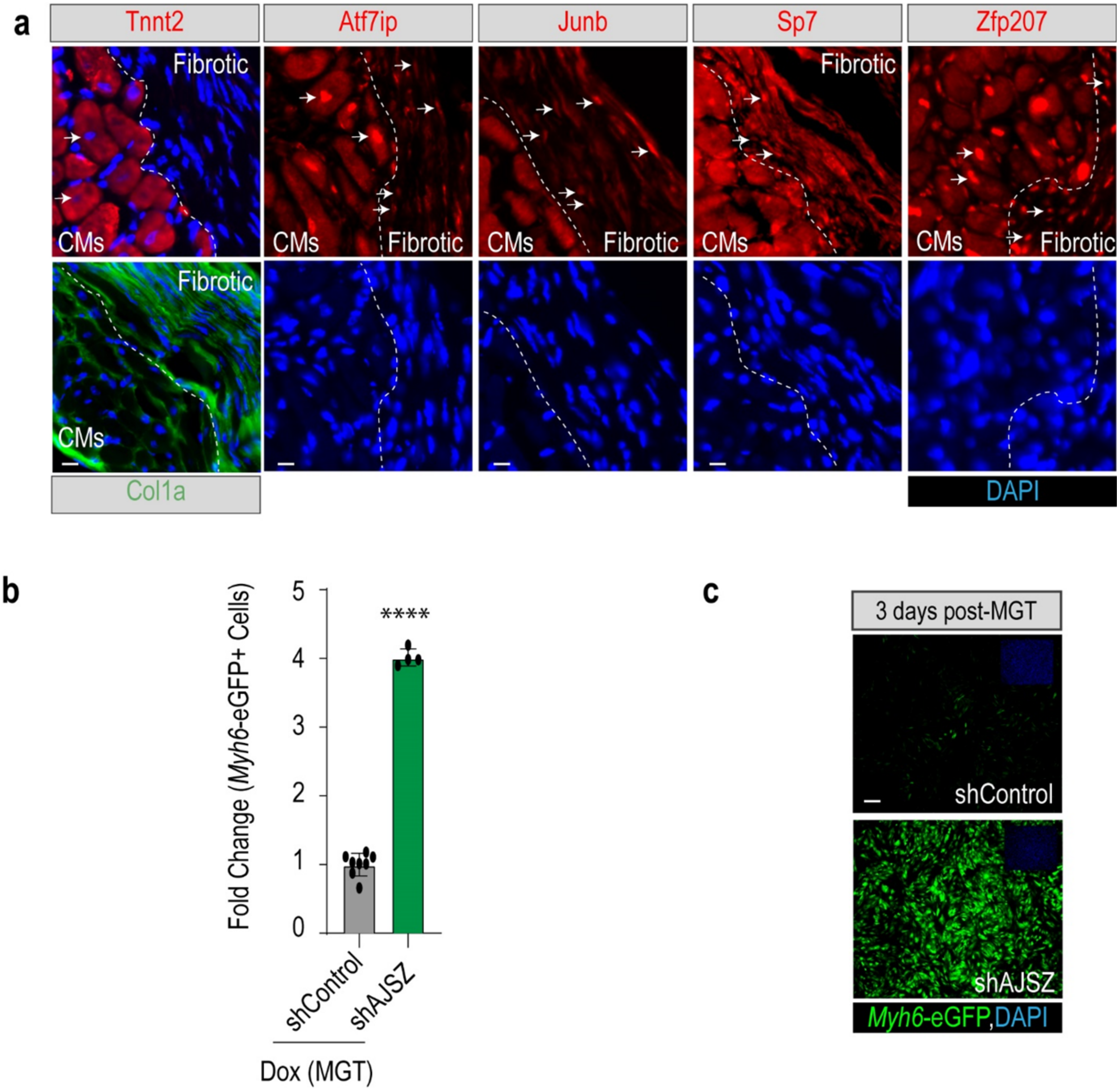

Supplemental Figure 11. (**a**) Quantification of CR efficiency (% of Myh6-eGFP cells) in shControl, n=8, vs shAJSZ, n=4, conditions in iMGT-MEF assay. Data are presented as mean values +/- standard deviation. (**b**) Representative images of shAJSZ- and shControl-infected iMGT-MEFs on day 3 after MGT induction. Scale bars: 50 µm. **(c)** Immunostaining of AJSZ, Tnnt2 (red) marking the cardiac compartment, and Col1 marking the fibrotic compartment, of

mouse infarcted heart at regions of injury, 4 weeks post-MI. Nuclei are stained with DAPI (blue). Scale bars: 10 μm. Student's t-test: *$p<0.05$, **$p<0.01$, ***$p<0.001$, ****$p<0.0001$.

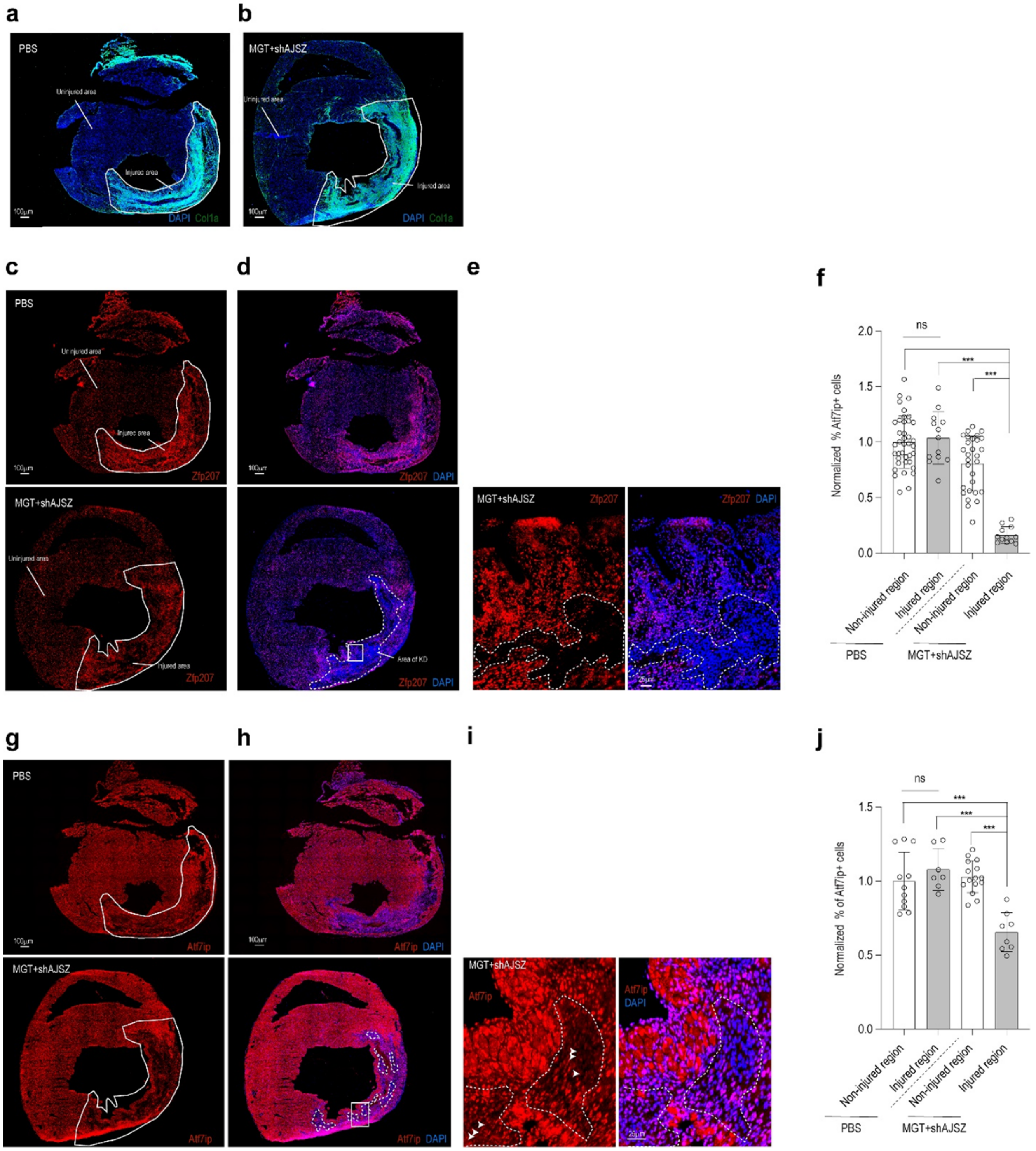

Supplemental Figure 12. (**a,b**) Representative images of Col1 (green) and DAPI (blue) immunostaining for both PBS and MGT+shAJSZ conditions, 4 days after echo-assisted injection. The injured area (= Col1+ region) is outlined with a solid white line. Scale bar is 100µm. (**c**) Representative images of Zfp207 Immunostaining for both PBS and MGT+shAJSZ conditions. The region outlined by the solid white line represents the injured area (=Col1+ area). (**d**) Same images as in **c**) with DAPI overlay. The region outlined by the dotted white line represents the area of Zfp207 KD. The white square marks the region magnified in **e**). (**e**) Example of a region containing a mixture of cells expressing high levels and low levels of Zfp207 (**f**) Histogram showing the quantification of Zfp207 signal in both injured and non-injured regions of PBS and MGT+shAJSZ conditions. Scale bar for **c** and **d** is 100µm. Scale bar for **e**) is 25µm. (**g**) Representative images of Atf7ip Immunostaining for both PBS and MGT+shAJSZ conditions. The region outlined by the solid white line represents the injured area (=Col1+ area). (**h**) Same images as in **g**) with DAPI overlay. The regions outlined by the dotted white line represent the area of Atf7ip KD. The white square marks the region magnified in **i**). (**i**) Example of a region containing a mixture of cells expressing high levels and low levels of Atf7ip j) Histogram showing the quantification of Atf7ip signal in both injured and non-injured regions of PBS and MGT+shAJSZ conditions. Scale bar for **g** and **h** is 100µm. Scale bar for **i**) is 25µm. Data in figure are presented as mean values +/- standard deviation. Student's t-test: *$p<0.05$, **$p<0.01$, ***$p<0.001$, ****$p<0.0001$.

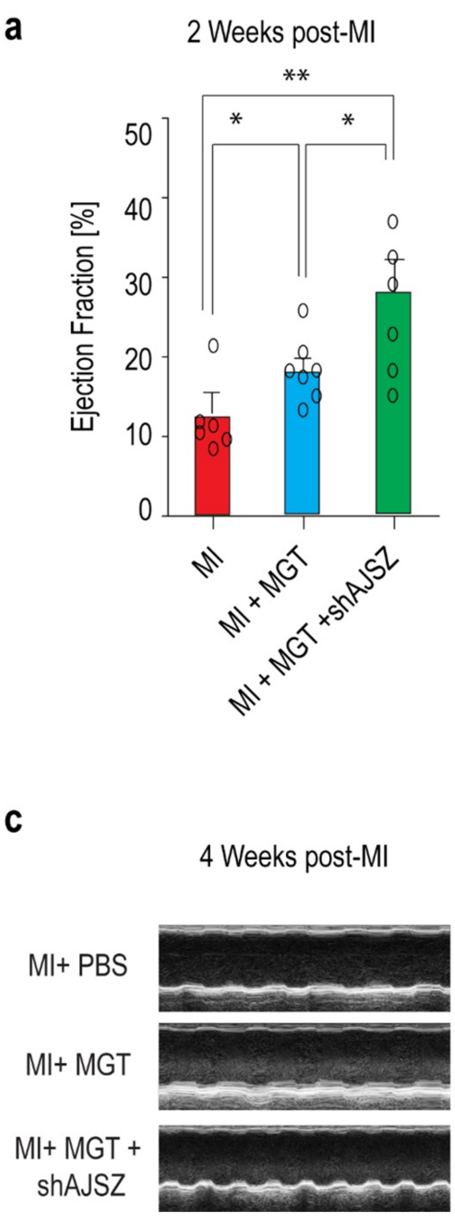

Supplemental Figure 13. (**a**) Ejection fraction (EF) and (**b**) fractional shortening (FS) of the left ventricle were serially quantified by echocardiography in mice injected with PBS, MGT and MGT+ shAJSZ 2 weeks after MI. For EF and FS quantification: PBS treated mice n=10, MGT treated mice n=13, and MGT+shAJSZ treated mice n=11. Data in figure are presented as mean values +/- standard deviation. Student's t-test: *p<0.05, **p<0.01, ***p<0.001, ****p<0.0001. Groups were compared using two-tailed unpaired. (**c**) Examples of echocardiograms from PBS, MGT or MGT+shAJSZ conditions are used to measure ventricular contractility 4 weeks after MI.

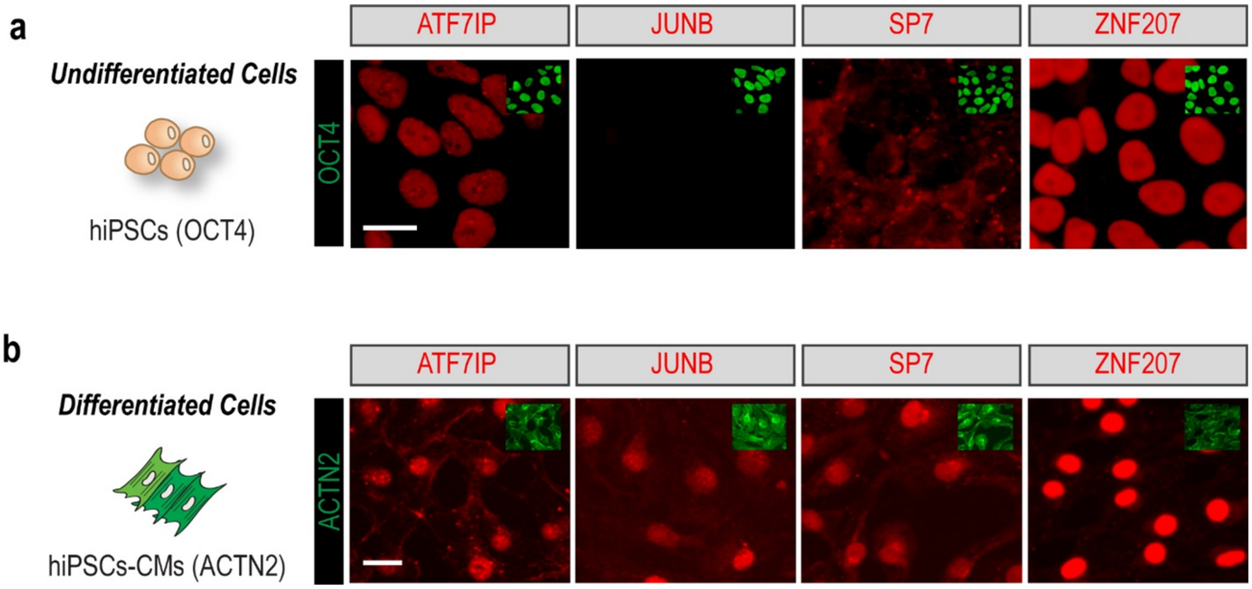

Supplemental Figure 14. (**a**) Immunostaining of ATF7IP, JUNB, SP7 and ZNF207 (red) and pluripotency marker OCT4 (green) in hiPSCs. (**b**) Immunostaining of AJSZ (red) and marker of cardiac differentiation ACTN2 (green). Nuclei are stained with DAPI (blue, top left insets). Scale bars: 30 µm. N=4 per condition. (a and b) schematics are modified from Cunningham, T. J. et al. Id genes are essential for early heart formation. Genes & development, doi:10.1101/gad.300400.117 (2017). - CC-BY 4.0.

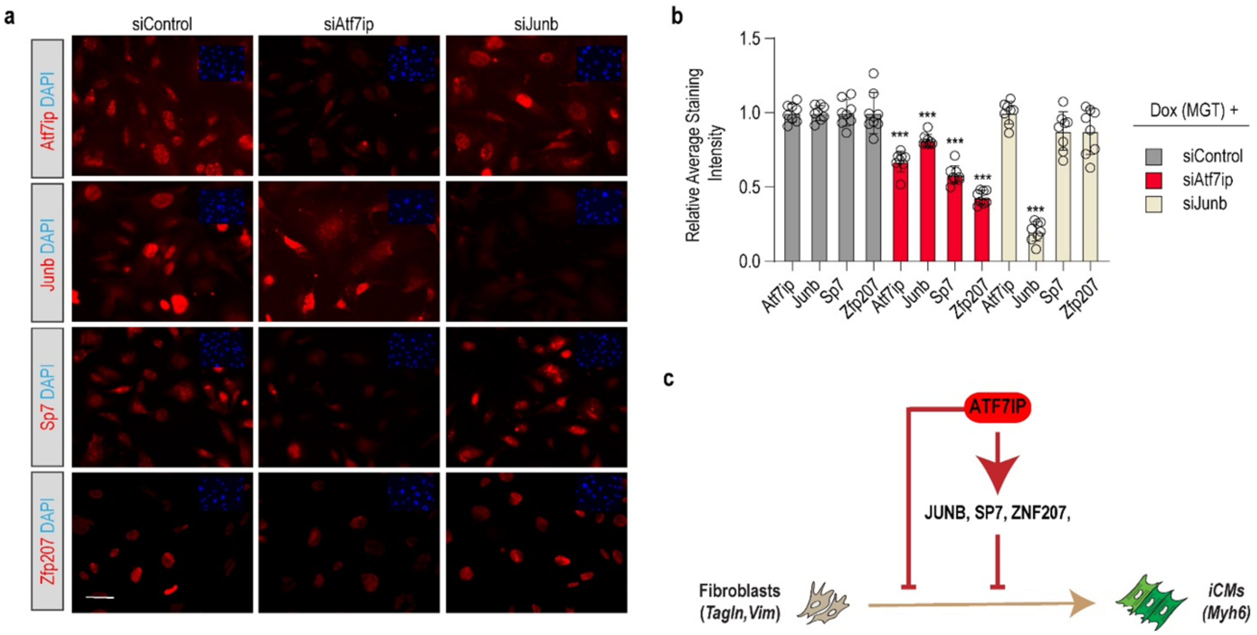

Supplemental Figure 15. (a) Representative immunofluorescence images of Atf7ip, Junb, Sp7 and Zfp207 protein levels (red) in response to siControl, siAtf7ip or siJunb, days 3 days post-transfection in iMGT-MEFs. DAPI is in blue. Scale bars: 50 µm. (**b**) Bar graph showing quantification of the average Atf7ip, Junb, Sp7 and Zfp207 staining intensity in response to siControl, siAtf7ip or siJunb transfection. N=8 per condition. Data are presented as mean values +/- standard deviation. ***P< 0.001. Groups were compared using two-tailed unpaired analysis. Scale bars: 50 µm. (**c**) Model depicting that Atf7ip acts upstream of Junb, Sp7 and Zfp207 by directly or indirectly controlling their proteins levels. (c) schematic is modified from Cunningham, T. J. et al. Id genes are essential for early heart formation. Genes & development, doi:10.1101/gad.300400.117 (2017). - CC-BY 4.0.
