# Supplementary material for: Conserved transcription factors promote cell fate stability and restrict reprogramming potential in differentiated cells
Source: Nat Commun. 2023 Mar 27;14:1709. doi: 10.1038/s41467-023-37256-8 (PMC10043290; doi:10.1038/s41467-023-37256-8)
Supplement: Supplementary file 3 — Description of Additional Supplementary Files [file 41467_2023_37256_MOESM3_ESM.pdf]

Description of Additional Supplementary Files

File Name: Supplementary Data 1
Description: Transcription Factor Screen results.

File Name: Supplementary Data 2
Description: Combination screen results.

File Name: Supplementary Data 3
Description: Annotated AJSZ ChIP-seq peaks.

File Name: Supplementary Data 4
Description: Open chromatin motifs.

File Name: Supplementary Data 5
Description: Open chromatin AJZ co-occupancy.

File Name: Supplementary Data 6
Description: AJZ open chromatin TSS and core promoter binding sites.

File Name: Supplementary Data 7
Description: Closed chromatin motifs.

File Name: Supplementary Data 8
Description: Closed AJS Co-occupancy.

File Name: Supplementary Data 9
Description: Differentiability accessible (open) chromatin at TSS for domain 2 and associated gene list and GO terms.

File Name: Supplementary Data 10
Description: domain 1 and 2 enriched TF binding motifs.

File Name: Supplementary Data 11
Description: List of genes associated with AJSZ binding (ChIP-seq) at core promoter regions.

File Name: Supplementary Data 12
Description: RNA-seq results.

File Name: Supplementary Data 13
Description: Differentially expressed genes bound by AJSZ (ChIP-seq) at core promoter regions.

File Name: Supplementary Data 14
Description: GO terms of differentially expressed genes bound by AJSZ at core promoter regions.

File Name: Supplementary Data 15
Description: Barriers screen results.

File Name: Supplementary Data 16
Description: Agonists screen results.

File Name: Supplementary Movie 1
Description: Movie showing calcium handling activity using Fluo-4 in reprogrammed iCMs at day 30 in MGT+siAJSZ condition.

File Name: Supplementary Movie 2
Description: Movie showing calcium handling activity using Fluo-4 in day 28 hPSC-CMs in siControl condition.

File Name: Supplementary Movie 3
Description: Movie showing calcium handling activity using Fluo-4 in day 28 hPSC-CMs in siAJSZ condition.
